# Self-regulated learning perception of undergraduate dental students during the COVID-19 pandemic: A nationwide survey in Brazil 

**DOI:** 10.4317/jced.58452

**Published:** 2021-10-01

**Authors:** Kamilla-Karla-Maurício Passos, Hélen-Kaline-Farias Bezerra, Augusto-César-Leal-da Silva Leonel, Flávia-Maria-Moraes Ramos-Perez, Hercílio Martelli-Júnior, Renato-Assis Machado, Paulo-Rogério-Ferreti Bonan, Danyel-Elias-da Cruz Perez

**Affiliations:** 1 PhD student, School of Dentistry, Universidade Federal de Pernambuco, Recife, Pernambuco, Brazil; 2 Graduate student, School of Dentistry, Universidade Federal de Pernambuco, Recife, Pernambuco, Brazil; 3 Professor, School of Dentistry, Department of Clinical and Preventive Dentistry, Universidade Federal de Pernambuco, Recife, Pernambuco, Brazil; 4 Professor, School of Dentistry, State University of Montes Claros, Montes Claros, Minas Gerais, Brazil; 5 Postdoctoral researcher, Piracicaba Dental School, State University of Campinas, Piracicaba, São Paulo, Brazil; 6 Professor, School of Dentistry, Universidade Federal da Paraíba, Joao Pessoa, Paraíba, Brazil

## Abstract

**Background:**

This study aimed to evaluate the perception of self-regulated learning of Brazilian undergraduate dental students during the COVID-19 pandemic.

**Material and Methods:**

A nationwide cross-sectional survey was conducted. Data were collected in 2020, through an anonymous self-administered virtual questionnaire, which comprised an initial section related to the students’ sociodemographic data, category of educational institution where they enrolled, and the possible impacts of COVID-19 pandemic on family income, teaching activities (maintained remotely or totally suspended), and self-perception of academic performance during e-learning. The second part comprising 31 questions related to the adapted Self-Regulated Learning Perception Scale (SRLPS). For statistical analysis, Student’s t-test of independent samples, Kruskal-Wallis, and Mann-Whitney U test were used, considering a significance of 5%.

**Results:**

From 779 students, 425 (54.6%) reported distance learning activities during the pandemic, and 354 (45.4%) experienced complete interruption of teaching activities. Students with good performance during e-learning were more skilled in self-regulated learning when compared to those who reported regular (*p* = 0.026), bad (*p* = 0.000), and very bad (*p* = 0.000) performance. In addition, students who stated a good performance during e-learning were more skilled in self-regulated learning than those with fully suspended teaching activities (*p* = 0.000).

**Conclusions:**

E-learning performance of undergraduate dental students during COVID-19 pandemic influenced the self-regulated learning perception. In addition, the pandemic negatively impacted the self-regulated learning of students who experimented full suspension of teaching activities. Changes in family’s income did not affect their self-regulated learning.

** Key words:**Dental education, community health, e-learning, learning, pandemics.

## Introduction

As a result of the COVID-19 pandemic, declared on March 11, 2020 by World Health Organization (1), several measures of distancing and social isolation have been imposed worldwide, to contain a further spread of the disease. Thus, it was necessary to apply closure policies in schools and universities worldwide (2). In April 2020, the inability to maintain face-to-face teaching activities impacted 91.2% of the world student population, according to United Nations Educational, Scientific and Cultural Organization (UNESCO). It is estimated that the suspension of these face-to-face activities may generate some disarrangement in the education process (3). Hence, e-learning has become a new routine for many students and teachers, but it still presents significant challenges to establish effectively. Currently, Brazil is experiencing a serious health crisis due to COVID-19. Since the beginning of the pandemic, the number of confirmed cases had reached 12,220,011 by March 26, 2021, with 300,685 deaths (1).

COVID-19 pandemic and the consequent emergency global isolation spurred a sudden need to diversify forms of learning (4). With little or any time to prepare for the transition from face-to-face to virtual teaching, teachers needed to reimagine how to proceed with the teaching-learning process, now remotely (5), as well as keep up with technological changes, learning theories and students’ needs (6). During this process, a greater effort has been necessary to continue teaching activities. However, this type of teaching activity requires adequate training that most Brazilian teachers are not equipped with (5-7).

The success of e-learning is influenced by political, cultural, economic aspects. The ease of access and use of these tools are important factors in this process (8). Moreover, the pandemic raises an important issue, which is the impact of isolation on the mental health of the population. It is important to highlight that anxiety and depression, in addition to uncertainties and increased information flow, will grow extensively and the negative consequences of this period of stress may manifest as a negative impact on learning (9). The impacts of prolonged social distance on mental health especially affect people aged 12 to 21 years old, a group composed mainly of students (10). Thus, students are usually part of the group of people who have a worsening mood, and it is possible that difficulties in adapting to life at home may have contributed to this characteristic being established and intensified during the pandemic (11).

Faced with these challenges, self-regulated learning consists of the student’s ability to plan and formulate educational goals and find ways to achieve them, using self-assessment strategies and time and resource management. Therefore, the students are active agents in their learning process. Students who practice self-regulation of learning are more proactive in this process and can monitor their behavior and exercise self-assessment (12,13). Students who have greater capacity for self-regulation tend to be more successful in learning and experience less stressful situations (14). The learning self-regulation ability allows to manage the emotions, thoughts and actions to deal with and adapt to higher education challenges. Therefore, the responsibility of teachers is to develop and optimize these skills in students (15). In relation to dentistry, the ability to reflect and evaluate learning is a fundamental component in patient care (16).

In a pandemic scenario, education changed dramatically. Therefore, the aim of this survey was to evaluate the perception of self-regulated learning of Brazilian undergraduate dental students during the COVID-19 pandemic.

## Material and Methods

The study was approved by the Research Ethics Committee of Federal University of Pernambuco (protocol 31721020.3.0000.5208) and complied with Declaration of Helsinki. It involves a nationwide cross-sectional survey of Brazilian undergraduate dental students. Data were collected between 15 June-15 July 2020, during the ongoing COVID-19 pandemic, through an anonymous self-administered virtual questionnaire hosted online (Google Forms, Google®).

The sample size calculation was performed by Epi Info software (CDC, USA), based on data from the latest survey on higher education in Brazil, which show 125,585 undergraduate dental students (17). Thus, it was estimated a minimum sample of 660 undergraduate students to ensure a 99% confidence level and a 5% margin of error.

The sample included 779 Brazilian dental students. The inclusion criteria were self-declared students enrolled in undergraduate dentistry courses in Brazil and that submitted their informed consent form before participation in the study. Students were recruited by e-mail, WhatsApp®, and social media (Instagram® and Facebook®).

The multiple-choice questionnaire contained an initial section with questions related to the participant’s demographic data, category of educational institution (public or private), the possible impacts of the COVID-19 pandemic on family income and college activities, and self-perception performance in e-learning during the pandemic. The second part consisted of 31 questions from the adapted Self-regulated Learning Perception Scale (SRLPS), a validated scale by Turan *et al*. (13). The questions were applied in Portuguese (Brazilian native language) according to the translation and validation by Barbosa *et al*. (15). In all questions, participants were asked about points of self-regulated learning perception and the responses were asked to represent that perception specifically during the COVID-19 pandemic.

In SRLPS, the questions are divided into four dimensions: 1) Motivation and action to learning; 2) Planning and goal setting; 3) Strategies for learning and assessment; 4) Lack of self-directedness. Cronbach’s alpha coefficient was used to measure the reliability of the instrument and the result was 0.93 for the total set and 0.77, 0.88, 0.89 and 0.84, for the first, second, third, and fourth dimensions, respectively.

For each question, there are five answer options according to the Likert Scale. Each answer can score on a five-point scale ranging from one to five. In the dimensions one, two, and three the score represents: 1- Strongly disagree; 2- Disagree; 3- Neither agree nor disagree; 4- Agree; 5- Strongly agree. However, for dimension 4, the numerical sequence corresponding to the score was inverted, considering that this dimension is related to negative characteristics in the learning process: 5- Strongly disagree; 4- Disagree; 3- Neither agree nor disagree; 2- Agree; 1- Strongly agree.

According to the established score, SRLPS generates a total score ranging from 31 to 155. Depending on the dimension, the score ranges from 6 to 30 for the first, 7 to 35 for the second, 11 to 55 for the third, and 7 to 35 for the fourth dimension. High scores indicate better perceived capacity for self-regulation of learning by the student, while lower scores represent less perceived capacity for self-regulation.

The responses for the variables were tabulated and analyzed using IBM SPSS Statistics software version 24.0 (IBM Corporation, New York, United States). Student’s t-test of independent samples was used to compare the means of the four dimensions and the total mean according to the variables “maintenance or suspension of teaching activities”, “category of educational institution (public or private)”, and “impact on family income”. Kruskal-Wallis test was performed to compare the mean scores on the variable “self-perception performance in e-learning” (excellent, good, regular, bad, very bad, and suspended activities). After these analyzes, the Mann-Whitney U test was performed to determine the different groups. The *p-value* ≤ 0.05 was taken as the limit for statistical significance.

## Results

A total of 779 Brazilian undergraduate dental students were included in the study. Most of them were females (n = 616 - 79.1%), and the mean age was 23.1 years (ranging from 18 to 60). Regarding the region of Brazil, 454 (58.3%) live in the Northeast, 155 (19.9%) in the Southeast, 89 (11.4%) in the Midwest, 51 (6.5%) in the North, and 30 (3.9%) in the South. For 492 (63.2%) students, the COVID-19 pandemic caused a decrease in their family income, whether 287 (36.8%) pointed no decrease in family income.

Of these students, 405 (52%) belonged to a public institution and 374 (48%) to a private institution. As a result of the COVID-19 pandemic and the need for social distancing and isolation, 425 (54.6%) students reported that the college continued with distance learning activities (e-learning) – 360 (84.7%) from private, and 65 (15.3%) from public institutions –, and 354 (45.4%) experienced a complete interruption of teaching activities – 340 (96%) from public, and 14 (4%) from private institutions. The group of students who experienced e-learning was asked about their perception of performance in this type of activity: 13 (2.8%) considered the performance excellent, 82 (17.6%) considered it good, 172 (37%) regular, 111 (23.9%) bad, and 87 (18.7%) very bad.

The total mean of the scores obtained by the Self-regulated Learning Perception Scale (SRLPS) was 113.7, and the standard deviation (SD) was 16.5. In analyzing the dimensions of the SRLPS separately, the average for the “Motivation and action to learning” dimension was 23.6 and SD was 3.3; the dimension “Planning and goal setting” obtained an average of 25.6 and SD 4.9; in “Strategies for learning and assessment”, the average was 40.3 and SD 6.7, and in “Lack of self-directedness”, the average was 24.3 and SD 5.5.

The association of SRLPS score means with the variables “maintenance or suspension of teaching activities”, “educational institution – public or private” and “impact on family income”, demonstrated that the students who continued their teaching activities through e-learning achieved better score only in the dimension “Lack of self-directedness” (*p* = 0.004). In addition, students from private institutions achieved better averages in the SRLPS total score (*p* = 0.003), and in the isolated dimensions of “Strategies for learning and assessment” (*p* = 0.006) and “Lack of self-directedness” (*p* = 0.000). Nevertheless, the impacts of the pandemic on students’ family income did not influence the average scores (Table 1).

**Table 1:**
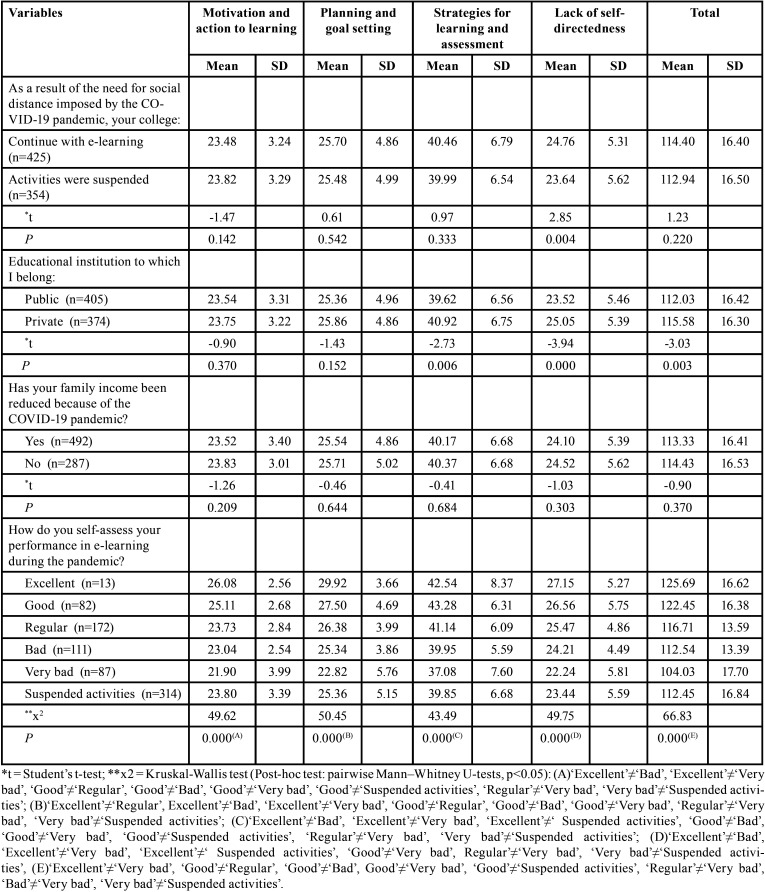
Students mean scores of self-regulated learning perception according to family income, college activities, category of educational institution (public or private), and self-perception in e-learning during the COVID-19 pandemic.

According to the performance in e-learning during the pandemic, it was observed that a better students’ perception of performance is associated with better ability to self-regulate learning according to the total SRLPS score. Students who reported excellent performance during e-learning were more skilled in self-regulated learning when compared to those who reported very bad performance (*p* = 0.001); the same effect was observed in the students with good performance compared to regular (*p* = 0.026), bad (*p* = 0.000), and very bad (*p* = 0.000) performance. In addition, students who stated a good performance during e-learning were more skilled in self-regulated learning than those with full suspended teaching activities (*p* = 0.000). Students who reported bad performance in e-learning presented better scores in total SRLPS when compared those with very bad performance (*p* = 0.022). Despite this, students who had total interruption of teaching activities presented better score on the total SRLPS compared to students who rated their performance as “very bad” during e-learning (*p* = 0.002). The associations of self-perceived performance at e-learning during the pandemic according to the self-regulating learning ability measured in each dimension of the SRLPS are shown in Table 1.

## Discussion

The COVID-19 pandemic and the need for social isolation caused changes in teaching activities at universities worldwide, as well as in the students’ routine. Thus, many students and teachers had to reformulate their teaching and learning practices, even amid a chaotic and unprecedented world scenario. Consequently, more than ever, students need to develop skills in handling emotions and behaviors to minimize the possible negative impacts of the pandemic on education. Given this, improved self-regulation skills can help students better cope with stressful situations (14). The ability to self-regulate learning can be a relevant characteristic for maintaining performance and achieving academic goals in the face of the COVID-19 pandemic.

The present study demonstrated that students who had a better self-perception of e-learning performance during the pandemic presented better self-regulation capacity measured by SRLPS when compared to the students with a worse perception of e-learning performance and students with full suspended teaching activities. The use of self-regulatory learning skills can improve students’ academic achievements, clinical performance, and mental well-being (18). Moreover, students’ self-regulatory capacity can be a performance indicator (19). Such considerations support our findings. However, it is important to consider that due to rigorous academic requirements, students may be dealing with issues such as anxiety, stress, and dissatisfaction and that the COVID-19 pandemic may contribute to intensifying these characteristics (11). Therefore, we consider that such issues can contribute to a worse perception of performance by the student. Some aspects, such as the student’s motivation and ability to get involved with self-directed learning tasks can influence the perception of performance (20). To deal with this situation, support strategies from family and friends can positively influence students’ focus and assist in completing tasks, enabling a reduction of stress (21). In contrast, Siddiqui and Khan (22) assume that moderate stress can direct students in the pursuit of self-regulation and concomitantly, the achievement of this skill can help them to deal with stressful situations.

In the same way, another question deserves consideration. Although the e-learning is already part of the teaching methodology of many higher education institutions around the world, and during the pandemic it is the only way to offer educational activities respecting social isolation (23), this is not a reality in most dental schools in Brazil. Thus, there was a need for abrupt migration to e-learning, which required rapid adaptation of teachers and students. This may explain the high percentage of students with perceived poor performance during e-learning, with consequent worse self-regulation capacity measured by SRLPS. There are not effective alternatives for full remote dental education (7). In Brazil, with the adoption of social inclusion policies, most dental students in Brazil come from low-income families, mainly in public institutions (7). This means that the access to adequate equipment and internet network that enable online teaching may be a difficulty for these students, resulting in probable bad perception of performance in e-learning (19). In addition, faced with these challenges, absence of teacher training and difficulty of internet access for many students, 45.4% of the participants in this study reported that they experienced a total interruption of the colleges’ teaching activities. These students also had a worse self-learning score when compared to students with a perception of good performance during e-learning. On the other hand, those who had suspension of all activities had scores of self-learning higher than those who had very bad perception of performance in e-learning. Thus, the student’s bad experience during e-learning is worse for self-learning than offering it. This finding reinforces the need for good planning for remote teaching. It should also be considered that not every student can adapt to this teaching modality.

Considering a dimension of SRLPS, students who experienced e-learning during the pandemic had a greater perceived capacity for self-regulation of learning observed in the dimension “lack of self-directedness”. This finding can be explained because distance learning makes the student the main author of their learning process (24). That is, teaching practices that encourage the student to actively conduct and monitor their own learning can improve the student’s self-directed ability (12). In the same way, students from private institutions showed a better perception of self-regulation of learning during the pandemic than public university students. This effect can demonstrate the importance of the continuity of teaching activities for the students’ self-regulation capacity, since in Brazil, most private institutions maintained classes remotely, as they are the ones that most have financial resources and conditions to offer this type of activity, in contrast to most Brazilian public universities.

In general, the respondents’ perception of self-regulated learning was high (113.7). In this regard, it is important to highlight that people tend to have an overestimated view of their cognitive skills (25) and hence, that number may not actually represent reality. This question highlights the fact that all data in this study are self-reported by the participants and this factor can act as a limitation of the study given the possible biases of recall and social desirability, which means the tendency to underreport socially undesirable attitudes and behaviors. Besides, other limitation should be considered as the recall bias that may explain the small disagreement between the number of students who reported the total suspension of teaching activities and those who commented on their experiences concerning distance education during the pandemic. Even so, such inconsistency is minimal and does not diminish the relevance of the data. In addition, the web-based convenience sample may result in some selection bias, with a given population being over- or under-represented. Nevertheless, considering the pandemic scenario, this is the only viable method for such surveys.

In conclusion, this study demonstrated that the self-regulated learning of undergraduate dental students during the COVID-19 pandemic was influenced by e-learning performance. There was a positive association between excellence/good self-perceived performance in e-learning during the pandemic and greater capacity for self-regulation of learning. In addition, the self-regulated learning was negatively influenced by the full suspension of teaching activities during the pandemic. These data reinforce the need to maintain well planned teaching activities in dental schools, even if remotely, contributing to the improvement of educational and academic objectives. Care to provide the student with a good experience during remote teaching activities is also essential. However, the impact of the pandemic on family income did not significantly affect the students’ ability to self-regulate learning. The data obtained by this study may be useful for the planning and decision making of dental schools regarding the development of teaching activities during the COVID-19 pandemic.
